# Patients’ acceptance of placebo antibiotics in Japan: a prescription for antimicrobial resistance

**DOI:** 10.1186/s40545-022-00470-8

**Published:** 2022-11-08

**Authors:** Atsushi Ito, Yuichi Watanabe, Takashi Okumura

**Affiliations:** 1 Faculty of Public Policy, Kyoto Prefectural University, Kyoto City, Kyoto Japan; 2 Faculty of Health and Welfare Services Administration, Kawasaki University of Medical Welfare, Kurashiki City, Okayama Japan; 3 School of Regional Innovation and Social Design Engineering, Kitami Institute of Technology, Kitami City, Hokkaido 090-8507 Japan

**Keywords:** Antimicrobial resistance, Ethical placebos, Antibiotic

## Abstract

**Background:**

The generalized use of antibiotics has led to the emergence of bacteria which are resistant to antimicrobial agents. This stems in part from the patient's tendencies to seek antibiotics for diseases when not necessary. Hence, this article investigated patient acceptance of prescribing placebos as a substitute for unnecessary antibiotics in Japan, where physicians are under severe time constraints and are unable to offer explanations and persuade patients who demand unnecessary antibiotics prescription.

**Methods:**

A web-based questionnaire was administered to assess patients’ acceptance of the placebo treatment under informed consent. One thousand participants representing all genders and age-class were randomly selected from the online panel of a web-survey company.

**Results:**

The results showed that 67.9% of the participants were “satisfied” to receive such treatments, whereas 20.6% indicated acceptance of the prescription but without satisfaction. In total, 88.5% of the participants accepted the prescription of placebo, a result consistent with that of a preceding study on placebo treatments conducted in the United States. In the survey, tone of persuasion did not affect the patients’ attitudes; however, patients who were loyal to their physicians exhibited lower refusal rates.

**Conclusion:**

The survey results showed that the prescription of “ethical placebos” could be an acceptable option for the patients in Japan. For ethical concerns, an additional literature survey was conducted and the result suggested that such a radical treatment option could be justified, provided that the prescription benefits patients and informed consent is properly obtained. Albeit it is impractical to use, because of ethical and operational concerns, it would be worth further investigation to ensure diversity in the countermeasures for antimicrobial resistance, a major public health threat nowadays.

**Supplementary Information:**

The online version contains supplementary material available at 10.1186/s40545-022-00470-8.

## Background

The discovery and development of antibiotics has rendered obsolete many infections that have plagued humankind. However, the generalized use of antibiotics has resulted in an increasing number of resistant bacteria. The growing number of multidrug-resistant bacteria can lead to intractable bacterial infections, resulting in a global public health issue [1]. One of the causes of this problem is the inappropriate prescription of antibiotics for diseases such as common cold and influenza that cannot be cured by antibiotics [2]. Antibiotics are unnecessary for patients with viral infections. In acute rhinosinusitis, antibiotics and placebo prescriptions showed almost equivalent outcomes [3]. Moreover, excessive use of antibiotics can have harmful effects on a patient’s intestinal flora.

However, some patients demand antibiotic prescriptions, and some doctors continue to prescribe them. For example, an investigation of general practitioners (GPs) in Australia showed that over half of the GPs had prescribed antibiotics in response to patient expectations [4]. In the context of the increasing sense of patients’ rights and the risk of lawsuits, medical providers might be responding excessively to patient demands.

Patients in Japan are reportedly less knowledgeable about antibiotics than the patients in the European Union, and many patients demand antibiotic prescriptions even for conditions that do not require such [5]. The lack of patient awareness of anti-microbial resistance (AMR) has been considered an underlying factor of the situation, and the government has developed a national action plan for AMR [6, 7]. However, such attempts have not yielded a decisive solution for patients seeking antibiotic prescriptions. An even more serious problem is the medical practice workload in Japan. The estimated number of patient consultations per doctor reaches up to 5,633 for a year, which is 2.5 times higher than the average of the Organisation for Economic Co-operation and Development (OECD) countries, which is 2,277 [8]. Therefore, outpatient clinical care places a significant burden on physicians in the country, and the amount of time that can be spent on each patient is severely limited. In such a situation, physicians find it difficult to secure enough time to offer explanations and persuade patients who demand unnecessary antibiotics because there is a high pressure on physicians to prescribe antibiotics [5].

Therefore, we conceptualized an impractical, but theoretically effective, method that allows physicians to reduce unnecessary prescription of antibiotics by prescribing a placebo instead of antibiotics, presupposing the extreme situation in Japan. To avoid ethical issues associated with prescribing placebos to patients who demand antibiotics, the prescription should be issued under informed consent. Additionally, such a placebo prescription should be made only when patients demand antibiotics although evidence indicates that the placebo outweighs antibiotics. This present study conducted an online survey to determine patient acceptance of such a radical treatment option. In this article, we first define the ethical placebo concept, referring to related works. Second, the methodology of the survey is shown, followed by the results. Then, analysis and discussion are provided. The last section concludes the article and suggests future directions.

### Related works

The prescription of a placebo could reduce the number of actual prescriptions while avoiding dissensions between physicians and patients. However, placebo prescription against a patient’s will is considered unethical. Hence, informed consent must be obtained from patients for the prescription of placebos. This may appear contradictory, as this will compromise the purpose of a placebo if the patients know the content of the prescription. Therefore, we propose obtaining prior consent by means of a notice (on-wall signage) in the institution, rather than obtaining consent at the time of prescription. Additional file 1: Appendix 1 further discusses the ethical issues in the proposal, which indicates that the placebos can be justified under certain conditions with appropriate informed consent.

Although placebos have primarily been used in clinical trials, physicians use various placebo-like techniques out of clinical necessity [9, 10]. A review of 22 studies from 12 countries revealed that 17–80% of the physicians engaged in placebo-like treatments, such as injection of saline [10]. Such acts have been investigated from various viewpoints in medical ethics and have been justified only when certain conditions are met, namely benefit of the patients, considering the advantages and disadvantages of prescribing placebos [11].

In this context, although several studies have investigated physicians’ opinions of placebos [9, 10], patient acceptance has received less attention. Hull performed one such study investigating how patients think about placebo treatments and whether they are willing to accept such a treatment option [12]. The study was performed in Northern California and targeted adult patients who had visited an outpatient clinic at least once in the 6 months prior to the study. The structured interviews were administered by phone, and eligible participants were requested to voice their opinions on four scenarios of placebo treatments, each of which featured different patients who sought doctors for specific reasons. Each interview took 20 min on average, and the participants were sent $20 gift cards afterward. In this setting, 53.4% of patients accepted the interview, and 78.1% of the responders accepted the use of placebos in general. Regarding placebo use for common cold, which resembles our situation, 65.6% accepted the placebo prescription. Nevertheless, results of opinion surveys can differ significantly depending on the contexts and the questionnaires. Besides, the elaborated interview asked hypothetical questions regarding others and not about the patients themselves. Further, the interview setting might have compromised the anonymity of the answers about the ethical issues. Accordingly, we investigated patient opinions on the placebos for unnecessary antibiotics, in different cultural backgrounds with differently phrased questions, ensuring anonymity.

## Methods

To address the aforementioned problems, the survey was administered through a web-survey company, Rakuten Insight, Inc., which has one of the largest online panels in Japan (2.2 million members as of April 2019). We recruited online monitors who agreed to participate in the survey and randomly sampled 1000 people as participants. The survey was administered from January 15 to 17, 2019, on their online survey platform, as described below. In this study, the inclusion criterion of prior visits to clinics was omitted because Japanese residents usually visit doctors 12.9 times a year, on average, which is three times higher than the number of the United States [8].

We used a questionnaire that included 22 questions. Its English translation, together with the detailed protocol, is provided in Additional file 2: Appendix 2. Based on our conjecture that the author and tone of the explanation could affect patient acceptance, we prepared four types of in-hospital notices and randomly assigned the participants into four groups to determine their levels of acceptance of each notice. Table 1 shows the authors of the four types of in-hospital notices and explanations of their prescription content. For Notice type 1, the author was the clinic director and the notice emphasized that the prescription was based on practice guidelines from the Ministry of Health. For Notice type 2, the author was an academic and the prescription was based on practice guidelines from the academic society. For Notice type 3, the author was the clinic director and the notice emphasized that the prescription was based on the prescription policy of the clinic, in a rational tone. Finally, for Notice type 4, the author was the clinic director and the notice included an appeal to the patients’ emotions while stating that the prescription was based on clinic policy. Hence, we investigated the type of authority and explanation tones that might help improve the acceptance rate of the paternalistic intervention of physicians in patient decision-making. To analyze the various factors that might affect patient attitudes, we included questions that could be categorized into four types: compliance (Q.12, Q.13, and Q.14), health literacy (Q.9, Q.10, and Q.19), health awareness (Q.5, Q.6, and Q.8), access to healthcare facilities (Q.3, Q.4, and Q.7), and basic attributes (Q.1 and Q.2). This study was approved by the Institutional Review Board for Research with Human Subjects at the Kitami Institute of Technology (Approval Number 1016).

**Table 1:** Authors of the in-hospital notices and explanations of the prescriptions within the texts

Notice type	Author	Explanation of the prescription
Type 1	Clinic director	Based on practice guidelines from the Ministry of Health
Type 2	Academic society	Based on practice guidelines from an academic society
Type 3	Clinic director	Explained in a rational tone as a prescription policy of the clinic
Type 4	Clinic director	Explained in an emotional tone as a prescription policy of the clinic

## Results

Additional file 3: Appendix 3 gives the counts of all the responses to the questionnaire. First, we analyzed the degree of patient approval of the proposed placebo prescriptions as described by in-hospital notices at a clinic (Table 2a). In response to the question, “What will you do when you see the notice above?”, 668 (67.9%) participants answered, “I am satisfied with the policy and accept the prescription that my physician provides” (Active approval). In addition, 203 (20.6%) subjects answered, “I am not satisfied with the policy; or I do not understand it, but accept the prescription that my physician provides” (Passive approval), whereas 113 (11.5%) subjects answered, “I would tell my physician that I am dissatisfied with the policy or that I do not consent” (Refusal). We excluded 16 questionnaires from the results of this question due to missing answers (others). Overall, the results showed that 88.5% of the subjects would accept placebos instead of antibiotics, whereas 11.5% would not consent to such prescriptions. These results are similar to those obtained by an investigation conducted in the USA [12], which had a significantly different cultural background.

**Table 2:** Answer summary to questions

(a) “What will you do when you see the notice above?”							
Notice type	Active approval		Passive approval		Refusal		*p* value
Type 1	155	63.3%	61	24.9%	29	11.8%	n.s
Type 2	177	71.4%	50	20.2%	21	8.5%	
Type 3	168	68.6%	45	18.4%	32	13.1%	
Type 4	168	68.3%	47	19.1%	31	12.6%	
Total	668	67.9%	203	20.6%	113	11.5%	

(b) “How would you consider visiting the medical institution that displayed such a notice?”							
Notice type	Continue visiting		Change, if possible		Never revisit		*p* value
Type 1	177	71.7%	62	25.1%	8	3.2%	n.s
Type 2	187	75.7%	51	20.6%	9	3.6%	
Type 3	171	69.8%	67	27.3%	7	2.9%	
Type 4	179	72.8%	53	21.5%	14	5.7%	
Total	714	72.5%	233	23.7%	38	3.9%	

Chi-squared test

*n.s.* not significant

As these responses concern attitudes toward the in-hospital notices, the content of such notices could have greatly affected the responses. Therefore, we compared the results of four different types of in-hospital notices. Notice type 2 received the highest (177 subjects; 71.4%) active approval, followed by Notice types 3 and 4 (168 subjects; 68.6%), which indicated the clinic director as the author. Notice type 1, which indicated that the prescription was based on guidelines from the Ministry of Health, had the lowest score (155 subjects; 63.3%). Meanwhile, 21 (8.5%), 29 (11.8%), 31 (12.6%), and 32 (13.1%) subjects indicated clear refusal, “I would tell my physician that I am dissatisfied with the policy or that I do not consent” for Notice types 2, 1, 4, and 3, respectively. However, the Chi-squared test indicated no statistically significant differences among these proportions of responses. Hence, our results did not prove whether author authority or the tone of expression altered patient attitudes toward ethical placebos.

Next, we analyzed whether displaying a policy regarding the prescription of placebos instead of antibiotics would affect the revisit behaviors of patients (Table 2b). In response to the question, “How would you consider visiting the medical institution that displayed such a notice?”, 714 subjects (72.5%) responded “I would continue to visit, as necessary” (Continue visiting), whereas 233 subjects (23.7%) responded “I would visit another hospital if possible” (Change, if possible) and 38 subjects (3.9%) responded “I would never again visit the hospital” (Never revisit). We excluded 15 questionnaires from the results of this question due to missing answers (others).

Because attitudes are formed as a response to in-hospital notices, the notice contents could also have affected the revisit behaviors. Therefore, we compared the responses according to the four types of in-hospital notices. Notice type 2 received the highest (187 subjects; 75.7%) active approval, followed by Notice types 4, 1, and 3 with responses from 179 (72.8%), 177 (71.7%), and 171 (69.8%) participants, respectively. Seven (2.9%), eight (3.2%), and nine (3.6%) subjects refused for Notice types 3, 1, and 2, respectively. The Chi-squared test indicated no statistically significant differences for this question between attitudes caused by the types of notice.

Next, we analyzed the impact of ethical placebos in patients who demanded antibiotics for the common cold, which was our original theme (Table 3). Among the 984 participants, 344 (35.0%) requested antibiotics (Table 3a). A total of 462 (47.0%) participants do not request antibiotics, and 178 (18.1%) chose “do not know” as their answer to the question. Among those requesting antibiotics, 233 approved the ethical placebos, indicating that 67.7% of patients who demanded antibiotics would accept this treatment option. “Passive approval” was indicated by 82 (23.8%) participants; thus, 91.5% approved the treatment option, at some level. Only 8.4% indicated rejection. This trend was consistent across groups regardless of their having requested antibiotics; this result indicates that ethical placebos may be an alternative for prescribing unnecessary antibiotics upon patient request. The revisit behaviors of patients (Table 3b) also demonstrated similar results: 71.7% of participants who requested antibiotics would continue visiting the clinic, 26.0% might change if possible, and 2.3% would never revisit. There were no significant differences between those who did and who did not request antibiotics.

**Table 3:** Approval and revisit behavior of patients who demand antibiotics for common cold

(a) Approval of ethical placebos							
Demands antibiotics for common cold	Active approval		Passive approval		Refusal		*p* value
Yes/relatively, yes	233	67.7%	82	23.8%	29	8.4%	n.s
No/relatively, no	313	67.7%	84	18.2%	65	14.1%	
Do not know	122	68.5%	37	20.8%	19	10.7%	

(b) Revisit behavior of patients							
Demands antibiotics for common cold	Continue visiting		Change, if possible		Never revisit		*p *value
Yes/relatively, yes	248	71.7%	90	26.0%	8	2.3%	n.s
No/relatively, no	336	72.4%	108	23.3%	20	4.3%	
Do not know	130	74.3%	35	20.0%	10	5.7%	

Chi-squared test

*n.s.* not significant

Evaluation of the correlations between placebo acceptance and patient attributes revealed no statistically significant differences in acceptance in relation to sex, age, or educational background (Table 4a).

**Table 4:** Approval and revisit behavior of patients explained by basic attributes

(a) Approval of ethical placebos							
Category/variable	Active approval		Passive approval		Refusal		*p* value
Sex							
Male	345	69.8%	94	19.0%	55	11.1%	n.s
Female	323	65.9%	109	22.2%	58	11.8%	
Age groups							
20s	123	62.8%	46	23.5%	27	13.8%	n.s
30s	128	65.3%	46	23.5%	22	11.2%	
40s	142	72.1%	37	18.8%	18	9.1%	
50s	136	68.0%	42	21.0%	22	11.0%	
Over > 60	139	71.3%	32	16.4%	24	12.3%	
Educational background							
Junior high school	12	60.0%	5	25.0%	3	15.0%	n.s
High school	208	68.6%	59	19.5%	36	11.9%	
Junior college	64	74.4%	16	18.6%	6	7.0%	
University	268	66.0%	89	21.9%	49	12.1%	
Graduate school	34	68.0%	9	18.0%	7	14.0%	
Vocational school	70	68.0%	25	24.3%	8	7.8%	
Technical college	12	80.0%	0	0.0%	3	20.0%	
Others	0	0.0%	0	0.0%	1	100.0%	

(b) Revisit behavior of patients							
Category/variable	Active approval		Passive approval		Refusal		*p* value
Sex							
Male	369	74.4%	106	21.4%	21	4.2%	n.s
Female	345	70.6%	127	26.0%	17	3.5%	
Age groups							
20s	133	66.5%	56	28.0%	11	5.5%	*
30s	128	66.0%	56	28.9%	10	5.2%	
40s	151	75.9%	38	19.1%	10	5.0%	
50s	147	73.9%	48	24.1%	4	2.0%	
Over > 60	155	80.3%	35	18.1%	3	1.6%	
Educational background							
Junior high school	12	63.2%	6	31.6%	1	5.3%	n.s
High school	224	73.9%	65	21.5%	14	4.6%	
Junior college	68	78.2%	17	19.5%	2	2.3%	
University	284	70.3%	108	26.7%	12	3.0%	
Graduate school	37	71.2%	11	21.2%	4	7.7%	
Vocational school	78	75.0%	23	22.1%	3	2.9%	
Technical college	11	73.3%	2	13.3%	2	13.3%	
Others	0	0.0%	1	100.0%	0	0.0%	

Chi-squared test

*p < 0.05; n.s., not significant

Moreover, the cross-analysis of the correlations between attributes and revisit behaviors after viewing in-hospital notices indicated no differences for sex or academic background (Table 4b). However, we observed a statistically significant difference for age, indicating that the motivation to continue attending the institution may increase with age.

### Analysis

The results of this study revealed that > 80% of patients accepted the use of placebos as a substitute for antibiotics. In contrast, 11.5% of all patients clearly rejected this method and 23.7% indicated that they would seek future treatment at another hospital or clinic next time. To clarify the problems associated with the proposed method, it is necessary to identify the reasons for the patients’ refusal to accept this method and to analyze the reasons why patients expressed a desire to seek treatment at another medical facility. Thus, we analyzed the factors that may explain why some patients did and other did not consent to the display of notices regarding the use of placebos in hospitals as well as the subsequent behavior of patients regarding their hospital visits.

#### ﻿Attitudes regarding in-hospital notice

First, we conducted a cross-analysis of the relationship between attitudes toward notices in the hospital and the four factors regarding the respondents: “Compliance”, “Health literacy”, “Health awareness”, and the “Access to healthcare facilities” (Table 5).

**Table 5:** Acceptance of the notice according to different variables

Category/variable	Active approval		Passive approval		Refusal		*p* value
Compliance							
Compliance with instructions for medication (Q.12)							
Complies	389	72.4%	98	18.2%	50	9.3%	**
Not always	279	62.4%	105	23.5%	63	14.1%	
Compliance with physician instructions (Q.13)							
Complies	616	69.8%	183	20.7%	83	9.4%	**
Not always/Do not know	52	22.2%	122	52.1%	60	25.6%	
Understanding of explanation by physicians (Q.14)							
Satisfied	484	74.2%	114	17.5%	54	8.3%	**
Not always satisfied	146	54.9%	72	27.1%	48	18.0%	
Health literacy							
Understanding of Antibiotics (Q.19)							
Good	320	69.0%	107	23.0%	37	8.0%	**
Poor	348	66.9%	96	18.5%	76	14.6%	
Attitude toward influenza vaccination (Q.10)							
Positive	240	66.7%	85	23.6%	35	9.7%	n.s
Negative	399	68.3%	112	19.2%	73	12.5%	
Neither/do not know	29	72.5%	6	15.0%	5	12.5%	
Utilization of medicine notebook (Q.9)							
Full use	371	70.0%	105	19.8%	54	10.2%	n.s
Not really	297	65.4%	98	21.6%	59	13.0%	
Health awareness							
Walking habit (Q.6)							
Yes/relatively, yes	379	69.0%	102	18.6%	68	12.4%	n.s
No/relatively, no	232	66.7%	82	23.6%	34	9.8%	
Neither/do not know	57	65.5%	19	21.8%	11	12.6%	
Awareness of health management (Q.5)							
Yes/relatively, yes	367	67.6%	111	20.4%	65	12.0%	n.s
No/relatively, no	236	69.4%	69	20.3%	35	10.3%	
Neither/do not know	65	64.4%	23	22.8%	13	12.9%	
Experience with the denial of recommended examinations and treatment (Q.8)							
Yes/relatively, yes	367	67.6%	111	20.4%	65	12.0%	n.s
No/relatively, no	236	69.4%	69	20.3%	35	10.3%	
Neither/do not know	65	64.4%	23	22.8%	13	12.9%	
Access to healthcare facilities							
Number of hospitals and clinics nearby (Q.3)							
> 10	207	69.0%	56	18.7%	37	12.3%	n.s
4–9	224	70.2%	58	18.2%	37	11.6%	
1–3	188	66.2%	71	25.0%	25	8.8%	
0	10	50.0%	8	40.0%	2	10.0%	
Neither none of the above/do not know	39	63.9%	10	16.4%	12	19.7%	
Frequency of hospital/clinic visits (Q.4)							
Visiting a specific facility periodically	268	70.0%	66	17.2%	49	12.8%	**
Visiting not periodically, but regularly	92	64.8%	34	23.9%	16	11.3%	
Visit convenient facilities as needed	259	69.8%	82	22.1%	30	8.1%	
Trying to avoid seeing a doctor	47	54.7%	21	24.4%	18	20.9%	
Easiness to take a sick leave (Q.7)							
Easy/relatively easy	377	69.6%	107	19.7%	58	10.7%	n.s
Not easy/relatively not easy	179	64.4%	65	23.4%	34	12.2%	
Neither/do not know	112	68.3%	31	18.9%	21	12.8%	

Chi-squared test

*p < 0.05, **p < 0.01; n.s., not significant

In regard to questions on “Compliance”, we found a significant difference in the degree of acceptance of the in-hospital notices between the group that felt compliance was important and the group that did not. The specific items for which a significant difference was found in this category were “Compliance with instructions for medication”, “Compliance with physicians’ instructions”, and “Understanding of physician explanation.” For example, the rate of acceptance of in-hospital notices was low in the group that did not always follow instructions, although those who tended to follow a physician’s instructions were more likely to consent to the use of placebos.

Next, we examined the responses to questions regarding “health literacy.” The non-consent rate among those with the correct perception of antibiotics was 8.0%, whereas the non-consent rate among those with mistaken perceptions was 14.6%, indicating that patients with mistaken perceptions of antibiotics had a higher non-consent rate. Nevertheless, no significant difference was found among respondents who did or did not demand “influenza vaccination” (Q.10) and who used their medicine notebook or not (Q.9).

Examination of the responses to the questions on “health awareness” revealed no significant differences in consent rates between the groups in terms of “awareness of health management” (Q.5), “walking habit” (Q.6), and “experience with denial of recommended examinations and treatments” (Q.8).

Upon examination of the responses to the questions regarding “access to healthcare facilities”, the consent rates were significantly higher among those who regularly visited a medical facility and those with a regular physician than the rate among those who avoid being examined at a medical institution. However, no difference was found in the consent rates regarding the “Number of hospitals and clinics nearby” (Q.3) and “Easiness to take a sick leave” (Q.7).

#### ﻿Motivation for subsequent hospital visit

Next, we performed cross-analysis to determine the relationship between the motivation for seeking medical care at medical facilities and the following four factors, “Compliance”, “Health literacy”, “Health awareness”, and “Access to healthcare facilities”, after having seen a notice regarding placebos (Table 6).

**Table 6:** Revisit behavior according to different variables

Category/variable	Continue visiting		Change, if possible		Never revisit		*p* value
Compliance							
Compliance with medication instructions (Q.12)							
Complies	522	56.4%	383	41.4%	20	2.2%	**
Not always	24	50.0%	17	35.4%	7	14.6%	
Compliance to physicians’ instructions (Q.13)							
Complies	662	75.1%	201	22.8%	19	2.2%	**
Not always/do not know	52	50.5%	32	31.1%	19	18.4%	
Understanding of physician explanations (Q.14)							
Satisfied	509	77.8%	130	19.9%	15	2.3%	**
Not always satisfied	169	63.8%	80	30.2%	16	6.0%	
Health literacy							
Understanding of antibiotics (Q.19)							
Good	349	75.5%	99	21.4%	14	3.0%	n.s
Poor	365	69.8%	134	25.6%	24	4.6%	
Attitude toward influenza vaccination (Q.10)							
Positive	257	72.0%	89	24.9%	11	3.1%	*
Negative	429	72.7%	139	23.6%	22	3.7%	
Neither	28	73.7%	5	13.2%	5	13.2%	
Utilization of medicine notebooks (Q.9)							
Full use	394	74.6%	122	23.1%	12	2.3%	*
Not really	320	70.0%	111	24.3%	26	5.7%	
Health awareness							
Walking habit (Q.6)							
Yes/relatively, yes	413	75.1%	117	21.3%	20	3.6%	n.s
No/relatively, no	245	70.4%	91	26.1%	12	3.4%	
Neither/do not know	56	64.4%	25	28.7%	6	6.9%	
Awareness of health management (Q.5)							
Yes/relatively, yes	397	73.0%	132	24.3%	15	2.8%	**
No/relatively, no	253	74.6%	73	21.5%	13	3.8%	
Neither/do not know	64	62.7%	28	27.5%	10	9.8%	
Experience with the denial of recommended examinations and treatment (Q.8)							
Yes/relatively, yes	102	73.4%	30	21.6%	7	5.0%	**
No/relatively, no	563	74.4%	174	23.0%	20	2.6%	
Neither/do not know	49	55.1%	29	32.6%	11	12.4%	
Access to healthcare facilities							
Number of hospitals and clinics nearby (Q.3)							
More than > 10	221	73.2%	69	22.8%	12	4.0%	**
4–9	234	73.1%	78	24.4%	8	2.5%	
1–3	207	73.4%	68	24.1%	7	2.5%	
0	13	68.4%	5	26.3%	1	5.3%	
Neither none of the above/do not know	39	62.9%	13	21.0%	10	16.1%	
Frequency of hospital/clinic visits (Q.4)							
Visiting a specific facility periodically	291	76.4%	80	21.0%	10	2.6%	**
Visiting not periodically, but regularly	107	74.8%	31	21.7%	5	3.5%	
Visit convenient facilities as needed	263	70.5%	98	26.3%	12	3.2%	
Trying to avoid seeing a doctor	52	60.5%	23	26.7%	11	12.8%	
Easiness to take a sick leave (Q.7)							
Easy/relatively easy	417	77.1%	110	20.3%	14	2.6%	**
Not easy/relatively not easy	180	64.7%	85	30.6%	13	4.7%	
Neither/do not know	117	70.5%	38	22.9%	11	6.6%	

Chi-squared test

*n.s.* not significant

*p < 0.05, **p < 0.01

Our examination of questions related to “Compliance” revealed a significant difference between the group that was likely to abide by a physician’s instructions and the group that was not likely to abide by a physician’s instructions regarding behaviors related to the hospital visit. This trend is clearly indicated in the result observed for the percentage of those who said that they would “not visit this hospital again”, which rose sharply from 2.2% to 14.6%. Q.13 showed that the respondents who “comply” to physicians’ instructions tended to continue their visit, although others did not. Q.14 showed that the respondents who were satisfied with physician explanations (Q.14) continued their visit more than those who were not always satisfied. Based on these findings, we conclude that those patients who followed instructions regarding medication and physician instruction and those who were always satisfied with the explanations they received showed a willingness to continue receiving medical care at the same medical institution even after seeing the placebo notices posted in the facility.

Our examination of questions related to health literacy did not indicate significant impact of the correct perception of antibiotics on their hospital visit behavior. The history of influenza vaccinations (Q.10) did not also affected their behavior, however, those who responded “I do not know/I am not sure” to the vaccination history indicated four times higher refusal rate. However, in terms of the general trend, health literacy did not appear to have a major effect on the desire to continue seeking medical care at the same medical facility.

Examination of the questions related to “health awareness” revealed that there was no difference among the respondents in terms of “walking habit”. However, “I do not know” group of the “awareness of health management” question (Q.5) indicated approximately 3 times higher refusal rate to their continued visit. Likewise, “I do not know” group of the “Experience with the denial of recommended examinations and treatment” question (Q.8) indicated two to four times higher refusal rate. Overall, participants who showed a high degree of health awareness were likely to continue undergoing treatment at the same facility, whereas those with a low degree of health awareness were more likely to seek treatment at another facility.

Questions related to the “access to healthcare facilities” included three questions. First, the “number of nearby medical facilities” (Q.3) indicated a general trend that the higher percentage of residents of communities with several medical facilities from which to choose would continue receiving medical care at the same facility, compared with the percentage of those living in areas without medical facilities. This result may look contradictory. However, residents of communities with choices in medical facilities selected their doctors on their own, whereas residents of communities without nearby medical facilities might be forced to visit a facility. This might be explained by the loyalty to the clinics, suggested by a previously mentioned finding. Examination of the questions related to how often respondents visited a medical facility (Q.4) revealed that those respondents who normally visited a medical facility regularly and have a regular physician had higher desires to continue visiting the same medical facility than that among those who did not visit medical facilities, consistent with the finding above. Finally, examination of the questions related to the ability to take a sick leave (Q.7) indicated that 77.1% of the respondents who said that it was easy and 64.7% of the respondents who said that it was not easy expressed the desire to continue visiting the same medical facility. This finding suggests that those respondents who experienced no hardships when they wanted to visit a medical facility were more likely to continue visiting the same medical facility.

#### ﻿Factors determining patient attitudes toward placebos

Finally, we performed a cross-analysis of the questions related to the degree of acceptance of the proposed method and questions related to subsequent hospital visits (Table 7). The results indicated that 92.5% of those who said they would continue visiting and who responded that they were satisfied with the hospital’s policy would accept the prescription (active approval), whereas 38.5% responded that they were not satisfied with the policy or did not understand it but would accept the physician’s prescription (passive approval) and 15.2% refused the prescription. However, examination of the percentage of those who said they would “change hospitals, if possible” indicated that 7.1% responded with active approval, 59.5% responded with passive approval, and 57.1% refused. These findings indicate that although at least 90% of those who were satisfied with the prescription for a placebo would continue receiving medical care at the same facility, ~ 30% of those who did not accept the policy, those who could not understand it, and those who were dissatisfied with the policy of prescribing placebos and would tell their physician that they do not consent to this type of prescription might seek treatment at another medical facility.

**Table 7:** Approval of the policy and revisit behaviors, a cross-analysis

	Continue visiting		Change, if possible		Never revisit		*p* value
Active approval	615	92.5%	47	7.1%	3	0.5%	**
Passive approval	77	38.5%	119	59.5%	4	2.0%	
Refusal	17	15.2%	64	57.1%	31	27.7%	

Chi-squared test

*p < 0.01

The fact that as many as 30% of patients might seek treatment at other medical facilities is an unacceptably high number. However, the fact that 90% of those who consented to the prescription of placebos said that they would continue to seek medical care at the same facility suggests that medical facilities may be able to efficiently screen patients who are well-matched to their policies. As shown in Table 2b, the decline from 27.3% to 20.6% according to the type of notice regarding the use of placebos suggests that there may be further declines in the non-consent ratio with further improvement of the notices. In the future, we would conduct a detailed analysis of the psychology and behaviors of patients who do not consent to such polices and of patients who seek medical care at other medical facilities.

## Discussion

In this study, we investigated the acceptance level of placebo prescriptions, instead of antibiotic prescription, as a method of reducing the unnecessary use of antibiotics. A previous study conducted in a Christian nation found that 78.1% of patients might approve of placebos in general clinical use and 65.6% as a substitute for antibiotics, using a hypothetical question [12]. We investigated patient opinions on placebos for unnecessary antibiotics in different cultural backgrounds with an improved study setting and confirmed that placebo prescriptions as a substitute for antibiotic prescription satisfied 67.9% of patients and could be accepted by 88.5% of them. Further, our study identified sub-classes of patients with higher acceptance rates: patients who exhibited compliance, who accepted the recommended examinations and treatments, who had high health literacy, who did not tend to refuse recommended tests and treatment, who periodically visited a certain medical institution, and who had family physicians. The results also indicated that subjects who did not meet the above criteria were likely to refuse consent to placebo prescriptions. Although Japanese patients may respond in similar way to any type of prescriptions, the impact of this study will not be compromised by such results.

### Advantages

It is more preferable for physicians to provide an explanation for not prescribing antibiotics compared with prescribing placebos. In Japan, however, time constraints are too severe for physicians to give detailed and educational comments for each prescription [13]. Even worse, Japanese are less knowledgeable about the appropriate use of antibiotics [5], and there is a high demand for antibiotic prescriptions. Hence, it is unrealistic for physicians in Japan to provide explanations for refusing to prescribe antibiotics to each patient.

In such situations, another option for patients who demand antibiotics i*s* “delayed prescription”, which lies between the prescription and non-prescription options. In this approach, a prescription is provided for antibiotics but the physician asks the patient to wait for a certain amount of time before they deliver the prescription to the pharmacy, hoping that the symptoms subside by that time. During the waiting period, patients may observe changes in their symptoms. Such prescriptions have been shown to be effective in reducing antibiotic use [14], but unfortunately, this prescription strategy is not highly evaluated by physicians or patients [15]. Although they result in greater satisfaction than the no antibiotics option, patient satisfaction is somewhat lower than that for an immediate prescription [14].

Ethical placebos can benefit patients by reducing unnecessary prescriptions that can harm the entire society; they also help save the physicians’ time spent on explanations and help retain patient satisfaction. The obligation to provide an explanation can be fulfilled by making it mandatory to provide an explanation of the prescription content at the next visit. When a patient’s condition does not require antibiotics, experiencing recovery without antibiotics by means of a placebo can have significant educational value to the patient, as delayed prescription can help avoid unnecessary antibiotic use [16]. It is also an ideal timing to provide educational intervention, when patients demand antibiotics.

### Disadvantages

The results of our analysis indicated that there were differences in acceptance levels between patients who had at least one medical institution near their residence and those who had none. If there are no other medical institutions near the one with the notice, patients might be forced to continue visiting it against their will. Thus, if there is a limited number of medical facilities in an area, placebo prescriptions could result in a significant breach of the patients’ right to self-determination in treatment.

To avoid this problem, it might be best to limit the use of ethical placebos to medical institutions in competitive urban environments. Patients with a reasonable number of choices in medical institutions can exercise their rights to self-determine their treatment by selecting another institution or by refusing a placebo prescription. Patients who do not periodically or regularly attend an institution might also be excluded from the approach because the physicians may fail to provide explanations afterward.

In this paper, we proposed a method of obtaining patient consent for the use of ethical placebos by means of an in-hospital notice. However, in reality, patients may not recognize such a notice. In addition to this, the fact that 18.1% of the subjects responded “do not know” when asked whether they would demand antibiotics for a common cold suggests that some patients found it difficult to understand the meaning of such in-hospital notices. Hence, to ensure the efficacy of the in-hospital notice, it is essential to further study how to promote awareness and understanding of the notice and its contents.

### Limitation

Ethical placebos are designed to maximize the welfare of both the patients and the society, which are under severe resource limitations. However, 11.5% of the participants refused to accept this method (Table 2a) and 23.7% stated that they would subsequently change hospitals if possible (Table 2b). Clearly, such a treatment should not compromise the patients’ rights, as we further discussed in Additional file 1: Appendix 1. Decisions of the patients who passively approve ethical placebos or deny consent warrant further study.

This survey on the acceptance of the intervention was based on responses obtained from a structured questionnaire. Therefore, the reasons for the acceptance or refusal of the patients are unknown. It remains as subject for future work to examine the differences in attitudes toward prescriptions other than placebo and the extent to which posterior explanations of ethical placebo can change patients’ attitudes. It is desirable to investigate the issues further, possibly with qualitative interviews.

## Conclusion

The emergence and spread of multidrug-resistant bacteria are major threats to public health. Although various campaigns have been initiated in Japan as countermeasures, none of the efforts have been able to regulate the unnecessary use of antibiotics, either for patients or prescribing physicians. The strategy of prescribing ethical placebos proposed in this paper might work in cases where the disadvantages of unnecessary prescription outweigh the advantages to reduce the number of unnecessary antibiotic prescriptions while maintaining patient satisfaction and easing physicians’ burden of providing explanations. However, various aspects of the ethical placebo are controversial, including ethical issues, technical issues in designing actual operations conforming to current health insurance systems, and, above all, patient acceptance.

At this point of time, it is unrealistic to put this proposal into actual practice. First, the prescription would be considered inappropriate, and further investigations on ethical issues are required. Second, there must be technical issues to realize the scheme under health insurance systems. Third, the psychology and behavior of patients who indicated their non-consent to placebos or intention to change their medical institutions must be investigated to clarify the problems associated with our proposal. Nevertheless, the acceptance by 67.9% participants deserves attention because the ethical placebos can deliver educational opportunities for the patients to accept the evidence-based approaches. Because multilateral interventions have been shown to be more effective as countermeasure than single interventions [1], it would worth further investigation to ensure the diversity in the countermeasure for antimicrobial resistance, a major public health threat nowadays.

## Supplementary Information

**Additional file 1: Appendix 1**. Discussion and literature survey on the qualifications for the ethical placebo.

**Additional file 2: Appendix 2**. Survey Protocol and Questionnaire used in the online survey (English translation).

**Additional file 3: Appendix 3**. Responses to the questionnaire used in the study.

## Data Availability

The datasets generated and/or analyzed during the current study are not publicly available due to the data policy submitted to the review board, but are available from the corresponding author on reasonable request.

## Abbreviation

AMR: Anti-microbial resistance
