# Supplementary material for: Patients’ acceptance of placebo antibiotics in Japan: a prescription for antimicrobial resistance
Source: J Pharm Policy Pract. 2022 Nov 8;15:79. doi: 10.1186/s40545-022-00470-8 (PMC9641938; doi:10.1186/s40545-022-00470-8)
Supplement: Supplementary file 1 — Additional file 1: Appendix 1. Discussion and literature survey on the qualifications for the ethical placebo. [file 40545_2022_470_MOESM1_ESM.pdf]

Appendix 1:

Qualifications for ethical placebos

Placebo in clinical medicine

Placebos have been used mainly in clinical trials. Physicians, however, may use various placebo-like techniques out of clinical necessity [1, 2]. For example, if a patient demands strong analgesics such as non-steroidal anti-inflammatory drugs (NSAIDs) for chronic pain, the prescribing physician will face a problem. Although it may appear ethical to prescribe as requested, the long-term use of NSAIDs would damage the patient's health through peptic ulcers. In such cases, placebo could be an option, resulting in a healthier condition while maintaining patient satisfaction. Foddy argued that if certain conditions are met, placebo prescription could be justified in clinical settings in modern medicine [3].

Several studies have investigated placebo prescriptions from the viewpoint of medical ethics and have placed placebos in a positive light. These studies were conducted because patient health can be compromised by excessive respect for the patients' rights for self-determination. When a patient demands antibiotics even if their physician considers them to be unnecessary or harmful, should the physician follow the patient's wishes? Alternatively, should physicians take a considerable amount of time to explain to the patient that such a prescription will not be given, or simply refuse to prescribe antibiotics and allow the doctor-patient relationship to deteriorate?

In clinical settings, physicians face such dilemmas in which prescribing a placebo cannot be simply judged as unethical. Accordingly, this appendix discusses the ethical issues pertaining to the ethical use of placebos and proposes the factors that qualify such a treatment option.

Justification of the ethical placebo

Nowadays, physicians have a duty to provide an explanation and obtain approval from their patients [4]. Failure to obtain approval violates the right to self-determination. Providing a prescription that goes against the patients' will cause ethical problems. Accordingly, informed consent is necessary for ethical placebos to uphold the patients' right to self-determination. However, for a placebo to be effective, the patient must not be aware of the medication being a placebo. This indicates that an explanation of the placebo at the time of prescription must be omitted. One method of approaching this problem is to display a notice, indicating that placebos might be prescribed at the medical institution. If patients are provided ample opportunities to refuse, these actions cannot be considered as paternalistic practices that infringe upon patients' rights.

Another rather fundamental issue is "whether physicians can engage in paternalistic intervention in response to demands that are irrational from a medical viewpoint." Medical paternalism is described as follows: "Paternalism, generally speaking, seeks to prevent these sorts of poor decisions and encourage good ones through coercion or influence" [5], and this could also potentially infringe upon patients' rights. In this context, forcible engagement of the intervention by physicians in the case of patients with incorrect knowledge or misunderstanding of the facts is called *selective paternalism*. Because shared decision making between physicians and patients is costly, such selective paternalism has been ethically justified [6].

In addition, the harm principle espoused by J.S. Mill, wherein freedom that causes trouble (harm) to other people (society) cannot be allowed, concludes that prescribing antibiotics in response to medically irrational demands should not be allowed as this might result in the emergence of resistant bacteria that could cause harm to society [7]. This decision is also supported by the scarcity of medical resources.

When discussing the prescription of unnecessary antibiotics, one must consider not only the issue of the patient's right to self-determination but also the question of how patients' decisions can negatively affect society. If it were possible to prescribe a placebo instead of unnecessary antibiotics, physicians could reduce the number of antibiotic prescriptions while maintaining patient satisfaction. This could be effective as a countermeasure against multidrug-resistant bacteria and result in long-term benefits for patients and society.

Factors qualifying placebo prescription

If the justification meets the universal demand of society, the prescription of placebos as described above could be socially accepted under certain conditions. In this section, we propose the conditions necessary for the placebo prescription to be ethical.

1. **Target**
   First, ethical placebos must target patients who demand antibiotics despite their physician having judged antibiotics to be unnecessary. While patients have the right to make irrational decisions for themselves, certain limitations are required if such decisions are disadvantageous for others. Utilizing placebos to avoid prescribing antibiotics could also result in mid- to long-term advantages to patients, as this could reduce the emergence of resistant bacteria.

2. **Evidence**
   Secondly, for ethical placebos to be prescribed instead of antibiotics, the advantages of the placebo must be objectively proven to outweigh the disadvantages. For example, patients requesting antibiotics for viral infections, such as common cold, would meet this requirement. This may also apply to acute rhinosinusitis patients, as no significant difference in outcomes was observed between patients prescribed antibiotics and those prescribed placebos [8]. Reducing antibiotic prescriptions in such cases is reasonable.

3. **Informed consent**
   Thirdly, informed consent must be obtained from patients for the prescription of placebos. For this purpose, we propose obtaining prior consent by means of a notice (signage) in the institution regarding placebo prescriptions rather than obtaining consent from each patient at the time of prescription. The patients have the right to refuse if they are dissatisfied or object to such policies. In our proposal, patients can refuse such placebos at any time.

4. **Educational opportunity**
   Finally, an ex-post explanation of the placebo prescription must be provided. Physicians must fulfill their duty of explaining their prescription content to the patient at the next visit, while observing the symptoms. At the expense of appearing optimistic, most of the patients can be expected to experience recovery without antibiotics. If the patients recover without antibiotics, it would provide a fundamental lesson not possible with traditional campaigns aimed at patients who demand antibiotics.

If these conditions are met, it will be possible for physicians faced with patients who demand antibiotics to reduce the prescription of unnecessary antibiotics while protecting their right to self-determination. Physicians will also be able to maintain patient satisfaction with medical care while reducing the burden associated with offering explanations to patients who demand antibiotics. This would make it possible to fulfill the interests of the physicians, patients, and society, in contrast to the situation in which physicians prescribe medically unnecessary antibiotics in response to patient demands.

Residual risks

When a placebo is prescribed, there is always the possibility that the symptoms will progress, resulting in health damage. In the present study, 20% of the participants responded as, "*I am not satisfied with the policy, or I do not understand it, but accept the prescription that my physician provides*"; thus, some of these patients might feel dissatisfied and request an apology or compensation in cases of symptom exacerbation. Accordingly, the prescribing physician is at risk of lawsuits if ethical placebos are used.

In Japan, as of 2017, 857 medical malpractice lawsuits had been filed, which is only 1/100th the number of such lawsuits filed in the US. However, the average screening period for each case is approximately two years [9], a length that highly burdens physicians. Cases related to internal medicine account for the largest proportion (24%) of lawsuits [10]. Clinicians face the risk of health damage resulting from placebo prescriptions and of breakdown of the doctor-patient relationship due to lawsuits. However, since physicians understand the risks associated with placebos, it is likely that they will only prescribe them in cases that can be objectively proven to be low-risk cases. Physicians will use the option within the extent of risk that they can afford, only in cases where they can justify the prescription considering their relationship with their patients. Even without such a radical option, clinicians are practicing medicine with various risks and prescribing medicines that can potentially harm patients because of adverse reactions. Compared to the current risks that physicians are taking in daily practice, prescribing placebos to patients for whom antibiotics are unnecessary is not a significant risk, although this does not mean the risk is nonexistent.

From the viewpoint of patients, however, they may feel uneasy about the possibility of physicians mistakenly prescribing a placebo when antibiotics are medically needed. Alternatively, there might be concerns about physicians prescribing unnecessary placebos in order to obtain prescription fees. However, physicians deliberately prescribed placebos in this proposal, whilst being aware of their responsibility if the symptoms ultimately worsened. Thus, physicians would prescribe placebos only when patients demand antibiotics for a condition that does not medically require antibiotics. In this situation, there is little incentive for physicians to easily or inconsiderately offer placebo prescriptions because there are residual risks.

Ethical prescription of placebos may be an emerging risk for clinicians, which can demotivate them to take the radical option. Accordingly, in the actual utilization of the option, establishment of a national compensation system that covers health damages associated with the prescription of ethical placebos might be needed, in addition to the amendment of the National Health Insurance System to accommodate the treatment option.

References

[1] Tilburt, J.C., Emanuel, E.J., Kaptchuk, T.J., Curlin, F.A., Miller, F.G.: Prescribing "placebo treatments": results of national survey of us internists and rheumatologists. BMJ **337**, 1938 (2008)

[2] Fässler, M., Meissner, K., Schneider, A., Linde, K.: Frequency and circumstances of placebo use in clinical practice-a systematic review of empirical studies. BMC medicine **8**(1), 15 (2010)

[3] Foddy, B.: A duty to deceive: placebos in clinical practice. The American Journal of Bioethics **9**(12), 4–12 (2009)

[4] World Medical Association: Declaration of lisbon on the rights of the patient. Adopted by the 34th World Medical Assembly, Lisbon, Portugal (1981)

[5] Sneirson, J.F.: Soft paternalism for close corporations: Helping shareholders help themselves. Wisconsin Law Review **2008**, 900–941 (2008)

[6] Drolet, B.C., White, C.L.: Selective paternalism. AMA Journal of Ethics **14**(7), 582–588 (2012)

[7] Mill, J.S.: On Liberty (Fourth Edition). Longmans, Green, Reader, and Dyer, ??? (1869)

[8] Garbutt, J.M., Banister, C., Spitznagel, E., Piccirillo, J.F.: Amoxicillin for acute rhinosinusitis: a randomized controlled trial. The Journal of the American Medical Association **307**(7), 685–692 (2012)

[9] Supreme Court of Japan: Statistics of Medical lawsuit cases (in Japanese). `http://www.courts.go.jp/saikosai/vcms_lf/29052601heikinshinri.pdf` (2018)

[10] Supreme Court of Japan: The number of medical services-related lawsuits in district court by type of clinical departments (in Japanese). `http://www.courts.go.jp/saikosai/vcms_lf/29052604sinryokakmoku.pdf` (2018)
