# Supplementary material for: Patients’ acceptance of placebo antibiotics in Japan: a prescription for antimicrobial resistance
Source: J Pharm Policy Pract. 2022 Nov 8;15:79. doi: 10.1186/s40545-022-00470-8 (PMC9641938; doi:10.1186/s40545-022-00470-8)
Supplement: Supplementary file 2 — Additional file 2: Appendix 2. Survey Protocol and Questionnaire used in the online survey (English translation). [file 40545_2022_470_MOESM2_ESM.pdf]

Survey Protocol

The survey was administered through a web-survey company, Rakuten Insight, Inc., which has one of the largest online panels in Japan (2.2 million members as of April 2019). We recruited online monitors, who agreed to participate in the survey and randomly sampled 1,000 people as participants, as follows.

The platform regularly sends emails to its registered monitors that ask them to login to the platform's list of open questionnaires, without showing the survey contents. Thereafter, the participants are given "screening questions" that test their suitability for the survey they select. In our survey, we excluded underage (19 years or younger) and overage (80 years or older) individuals. We also excluded healthcare professionals to avoid unexpected biases. Next, the participants were given the survey outline and their consent was sought before they started the survey. The listing ended when the number of participants reached the predefined volume for each sex and age-class. The response rate comparable with usual mail-in surveys is not defined because of the nature of the online platform designed to avoid selection bias for both the organizer and the participants. The entire survey took ~10 minutes for each participant to complete.

In the questionnaire, questions that could identify individuals through their name, exact age, address, or medical history were not included. Thus, although online marketing firms may have private information on the participants, the survey was conducted anonymously and the authors received only anonymized data. There was no direct contact between the authors and the participants and none of the questions infringed upon their human rights. Consent to participate in the study was obtained by asking the participants whether they wished to participate after explaining the study purpose and details at the beginning of the questionnaire.

Questionnaire used in the survey

This is a survey to investigate patient requests for prescriptions at medical institutions, to clarify the realities, and to envisage an ideal future. The questionnaire does not contain questions about your privacy, and all responses will be statistically processed for further analysis. Answering these questions will be considered as consent to participation in the survey. The survey takes approximately 10 minutes to complete. We appreciate your cooperation.

Question 1. Choose your educational background.

1) Junior high school 2) High school 3) Junior college
4) University 5) Graduate school 6) Vocational school
7) Technical college 8) Others

Question 2. In which prefecture do you live?

Question 3. How many hospitals and clinics exist in your vicinity?

1) More than 10 2) 4-9 3) 1-3
4) 0 5) Don’t know / Neither of above

Question 4. How often do you visit a hospital or a clinic? Please choose the single most applicable answer.

1) I visit a specific facility periodically
2) I do not visit periodically, but regularly see a family physician
3) I visit convenient facilities as needed
4) I try to avoid seeing a doctor, even if necessary
5) Others

Your views on medicine and medical care

Question 5. Are you managing your body weight and trying not to eat too much?

1) Yes / Relatively, Yes 2) No / Relatively, No
3) Don’t know / Neither

Question 6. For your health, are you trying to walk as much as you can?

1) Yes / Relatively, Yes 2) No / Relatively, No
3) Don’t know / Neither

Question 7. Is it easy for you to take a sick leave and a vacation?

1) Easy / Relatively easy 2) Not easy / Relatively not easy
3) Don’t know / Neither

Question 8. Have you ever declined a medical examination or a treatment that your physicians or nurses recommended?

1) Yes / Relatively, Yes
2) No / Relatively, No
3) Don’t know / Neither

Question 9. Do you regularly use your medicine notebook?

1) Using quite often
2) Using regularly
3) Not really
4) Not using at all / I don’t have one.
5) Don’t know / None of above

Question 10. Do you get a flu shot?

1) Yes / Relatively, Yes
2) No / Relatively, No
3) Don’t know / Neither

Question 11. Do you request that your doctor prescribe antibiotics if you have symptoms like those of a common cold? Have you ever requested antibiotics?

1) Yes / Relatively, Yes
2) No / Relatively, No
3) Don’t know / Neither

Question 12. When you get sick, do you take the prescribed medicine as instructed by your doctor?

1) Always follow the instruction given
2) Mostly follow the instruction, but sometimes forget to take necessary doses, or do not follow the instruction
3) Mostly does not follow
4) Don’t know / Neither

Question 13. When you get sick, do you think you should take the medical treatment that your doctor recommends or advises?

1) Agree
2) Partially agree
3) Disagree
4) Strongly disagree
5) Don’t know / None of above

Question 14. Have you ever experienced an instance in which you were not satisfied with the explanation of the medications that your doctor gave you?

1) Always unsatisfied
2) Sometimes unsatisfied
3) Mostly satisfied
4) Always satisfied
5) Don’t know / Neither

An in-hospital notice about prescription policy

Suppose that you visited a medical institution with symptoms like those of a common cold. In the facility, you found signage describing the prescription policy of the doctor.

In-hospital notice Type 1[i]

In this clinic, we will not prescribe antibiotics when they are considered to be ineffective and could be harmful due to side effects, based on a guideline established by the Ministry of Health, Labor, and Welfare. Instead, we may prescribe medications that look like antibiotics, without side effects, with the aim of easing your anxiety and associated symptoms.

- If you do not agree with our prescription policy, please let us know.
- To avoid unnecessary bias, we do not tell you the type of medication at prescription
- We will explain the type of prescription at your next visit

Kazunari Sakaguchi, M.D.
Director, Sakaguchi Clinic

In-hospital notice Type 2

The clinical practice guidelines of our society do not recommend the prescription of antibiotics when they are considered to be ineffective and could be harmful due to side effects. This institution is an accredited facility of the society and follows the practice guideline that avoids the unnecessary prescription of antibiotics. Instead, the facility may prescribe medications that look like antibiotics, without side effects, with the aim of easing your anxiety and associated symptoms.

- If you do not agree with our prescription policy, please let us know.
- To avoid unnecessary bias, we do not tell you the type of medication at prescription
- We will explain the type of prescription at your next visit

Practice Guidelines Committee
The Japanese Society of Medical Practice

In-hospital notice Type 3

In this clinic, we will not prescribe antibiotics when they are considered to be ineffective and could be harmful due to side effects. Instead, we may prescribe medications that look like antibiotics, without side effects, with the aim of easing your anxiety and associated symptoms.

- If you do not agree with our prescription policy, please let us know.
- To avoid unnecessary bias, we do not tell you the type of medication at prescription
- We will explain the type of prescription at your next visit

Kazunari Sakaguchi, M.D.
Director, Sakaguchi Clinic

In-hospital notice Type 4

Antibiotics have made many infectious diseases treatable. However, the popularized use of antibiotics has resulted in the emergence of many bacteria that are resistant to antibiotics. Consequently, there are fewer and fewer effective antibiotics for infectious diseases and our descendants in need may lose any treatment option. The number of deaths from antimicrobial-resistant bacteria is expected to increase to as many as ten million in 2050. Accordingly, in our clinic, we will not prescribe antibiotics when they are considered to be ineffective and could be harmful due to side effects. Instead, we may prescribe medications that look like antibiotics, without side effects.

- If you do not agree with our prescription policy, please let us know.
- To avoid unnecessary bias, we do not tell you the type of medication at prescription
- We will explain the type of prescription at your next visit

Kazunari Sakaguchi, M.D.
Director, Sakaguchi Clinic

Question 15. What will you do when you see the notice above?

1) I am satisfied with the policy and accept the prescription that my physician provides
2) I am not satisfied with the policy; or, I do not understand it but, accept the prescription that my physician provides
3) I would tell my physician that I am dissatisfied with the policy or that I do not consent
4) Others

Question 16. How would you consider visiting a medical institution that displayed such signage?

1) I would continue to visit, as necessary
2) I would visit another hospital if possible
3) I would never again visit the hospital
4) Others

Question 17. This question is for those who would not revisit the hospital. Would you change your decision if the bill were cheaper?

1) If 2,000 yen becomes 1,500 yen, my decision may change
2) If 2,000 yen becomes 1,000 yen, my decision may change
3) If 2,000 yen becomes 500 yen, my decision may change
4) My decision may not change
5) Others

Question 18. This question is for those who would not revisit the hospital. Please choose one reason that matches your case.

1) Because I still want antibiotics to be prescribed
2) Because I cannot have prior explanation about the prescription
3) Because I am not satisfied nor convinced by the notice.
4) Because I cannot accept the attitude to omit the explanation
5) Others

Question 19. The popularization of antibiotics may cause various effects. Please choose all that apply to your understanding.

1) It reduces death and exacerbation due to bacterial infection
2) It reduces death and exacerbation due to viral infection
3) Antimicrobial-resistant bacteria may emerge
4) It is profitable for pharmaceutical companies
5) Pharmacy and medical institutions may profit
6) It leads to a cleaner and more hygienic environment
7) Others
8) I don’t know

Question 20. If a patient requests the prescription of a drug that is not effective for treatment, the doctor may refuse to prescribe. For what kinds of diseases would you accept such policy? Please choose all that apply.

1) Common cold 2) Headache 3) Abdominal pain
4) Backache 5) Others
6) Cannot accept for any disease

Question 21. Have you ever heard of the word “placebo”?

1) Yes, and I can explain the meaning of the word
2) Yes, I have heard of the word, but I cannot explain
3) I have not heard of the word / I don’t know.

Question 22. Please let us know if you have any comments on the notice for prescription policy.

---

[i] After the survey, a minor typographic error was found in this notice (Type 1), which might have some impact on the acceptance by participants.
