# Supplementary material for: Patients’ acceptance of placebo antibiotics in Japan: a prescription for antimicrobial resistance
Source: J Pharm Policy Pract. 2022 Nov 8;15:79. doi: 10.1186/s40545-022-00470-8 (PMC9641938; doi:10.1186/s40545-022-00470-8)
Supplement: Supplementary file 3 — Additional file 3: Appendix 3. Responses to the questionnaire used in the study. [file 40545_2022_470_MOESM3_ESM.pdf]

Responses to the questionnaire

Q1. Choose your educational background.

	n	%
1) Junior high school	20	2.0
2) High school	309	30.9
3) Junior college	88	8.8
4) University	410	41.0
5) Graduate school	53	5.3
6) Vocational school	104	10.4
7) Technical college	15	1.5
8) Others	1	0.1
Total	1000	100

Q2. In which prefecture do you live?

		n	%
1)	Hokkaido	51	5.1
2)	Aomori	9	0.9
3)	Iwate	8	0.8
4)	Miyagi	18	1.8
5)	Akita	5	0.5
6)	Yamagata	4	0.4
7)	Fukushima	9	0.9
8)	Ibaraki	18	1.8
9)	Tochigi	7	0.7
10)	Gunma	12	1.2
11)	Saitama	66	6.6
12)	Chiba	41	4.1
13)	Tokyo	143	14.3
14)	Kanagawa	75	7.5
15)	Niigata	15	1.5
16)	Toyama	10	1.0
17)	Ishikawa	6	0.6
18)	Fukui	5	0.5
19)	Yamanashi	2	0.2
20)	Nagano	13	1.3
21)	Gifu	16	1.6
22)	Shizuoka	34	3.4
23)	Aichi	65	6.5
24)	Mie	15	1.5
25)	Shiga	15	1.5
26)	Kyoto	32	3.2
27)	Osaka	85	8.5
28)	Hyogo	57	5.7
29)	Nara	9	0.9
30)	Wakayama	9	0.9
31)	Tottori	4	0.4
32)	Shimane	7	0.7
33)	Okayama	20	2.0
34)	Hiroshima	22	2.2
35)	Yamaguchi	9	0.9
36)	Tokushima	6	0.6
37)	Kagawa	5	0.5
38)	Ehime	9	0.9
39)	Kochi	2	0.2
40)	Fukuoka	26	2.6
41)	Saga	2	0.2
42)	Nagasaki	6	0.6
43)	Kumamoto	9	0.9
44)	Oita	4	0.4
45)	Miyazaki	1	0.1
46)	Kagoshima	7	0.7
47)	Okinawa	7	0.7
48)	Others	0	0.0
	Total	1000	100

Q3. How many hospitals and clinics exist in your vicinity?

	n	%
1) More than 10	309	30.9
2) 4-9	323	32.3
3) 1-3	286	28.6
4) 0	20	2.0
5) Don’t know / Neither of above	62	6.2
Total	1000	100

Q4. How often do you visit a hospital or a clinic? Please choose the single most applicable answer.

	n	%
1) I visit a specific facility periodically	388	38.8
2) I do not visit periodically, but regularly see a family physician	145	14.5
3) I visit convenient facilities as needed	379	37.9
4) I try to avoid seeing a doctor, even if necessary	86	8.6
5) Others	2	0.2
Total	1000	100

Q5. Are you managing your body weight and trying not to eat too much?

	n	%
1) Yes / Relatively, Yes	553	55.3
2) No / Relatively, No	343	34.3
3) Don’t know / Neither	104	10.4
Total	1000	100

Q6. For your health, are you trying to walk as much as you can?

	n	%
1) Yes / Relatively, Yes	559	55.9
2) No / Relatively, No	353	35.3
3) Don’t know / Neither	88	8.8
Total	1000	100

Q7. Is it easy for you to take a sick leave and a vacation?

	n	%
1) Easy / Relatively easy	551	55.1
2) Not easy / Relatively not easy	281	28.1
3) Don’t know / Neither	168	16.8
Total	1000	100

Q8. Have you ever declined a medical examination or a treatment that your physicians or nurses recommended?

	n	%
1) Yes / Relatively, Yes	142	14.2
2) No / Relatively, No	768	76.8
3) Don’t know / Neither	90	9.0
Total	1000	100

Q9. Do you regularly use your medicine notebook?

	n	%
1) Using quite often	294	29.4
2) Using regularly	243	24.3
3) Not really	254	25.4
4) Not using at all / I don't have one.	193	19.3
5) Don't know / None of above	16	1.6
Total	1000	100

Q10. Do you get a flu shot?

	n	%
1) Yes / Relatively, Yes	364	36.4
2) No / Relatively, No	596	59.6
3) Don't know / Neither	40	4.0
Total	1000	100

Q11. Do you request that your doctor prescribe antibiotics if you have symptoms like those of a common cold? Have you ever requested antibiotics?

	n	%
1) Yes / Relatively, Yes	350	35.0
2) No / Relatively, No	465	46.5
3) Don't know / Neither	185	18.5
Total	1000	100

Q12. When you get sick, do you take the prescribed medicine as instructed by your doctor?

	n	%
1) Always follow the instruction given	546	54.6
2) Mostly follow the instruction, but sometimes forget to take necessary doses, or do not follow the instruction	400	40.0
3) Mostly does not follow	27	2.7
4) Don't know / Neither	27	2.7
Total	1000	100

Q13. When you get sick, do you think you should take the medical treatment that your doctor recommends or advises?

	n	%
1) Agree	416	41.6
2) Partially agree	480	48.0
3) Disagree	49	4.9
4) Strongly disagree	11	1.1
5) Don't know / None of above	44	4.4
Total	1000	100

Q14. Have you ever experienced an instance in which you were not satisfied with the explanation of the medications that your doctor gave you?

	n	%
1) Always unsatisfied	25	2.5
2) Sometimes unsatisfied	244	24.4
3) Mostly satisfied	440	44.0
4) Always satisfied	221	22.1
5) Don't know / Neither	70	7.0
Total	1000	100

Q15. What will you do when you see the notice above?

	n	%
1) I am satisfied with the policy and accept the prescription that my physician provides	668	66.8
2) I am not satisfied with the policy; or, I do not understand it but, accept the prescription that my physician provides	203	20.3
3) I would tell my physician that I am dissatisfied with the policy or that I do not consent	113	11.3
4) Others	16	1.6
Total	1000	100

Q16. How would you consider visiting a medical institution that displayed such signage?

	n	%
1) I would continue to visit, as necessary	714	71.4
2) I would visit another hospital if possible	233	23.3
3) I would never again visit the hospital	38	3.8
4) Others	15	1.5
Total	1000	100

Q17. This question is for those who would not revisit the hospital. Would you change your decision if the bill were cheaper?

	n	%
1) If 2,000 yen becomes 1,500 yen, my decision may change	20	7.4
2) If 2,000 yen becomes 1,000 yen, my decision may change	71	26.2
3) If 2,000 yen becomes 500 yen, my decision may change	58	21.4
4) My decision may not change	119	43.9
5) Others	3	1.1
Total	271	100

Q18. This question is for those who would not revisit the hospital. Please choose one reason that matches your case.

	n	%
1) Because I still want antibiotics to be prescribed	29	10.7
2) Because I cannot have prior explanation about the prescription	118	43.5
3) Because I am not satisfied nor convinced by the notice.	59	21.8
4) Because I cannot accept the attitude to omit the explanation	57	21.0
5) Others	8	3.0
Total	271	100

Q19. The popularization of antibiotics may cause various effects. Please choose all that apply to your understanding.

	n	%
1) It reduces death and exacerbation due to bacterial infection	505	50.5
2) It reduces death and exacerbation due to viral infection	470	47.0
3) Antimicrobial-resistant bacteria may emerge	420	42.0
4) It is profitable for pharmaceutical companies	176	17.6
5) Pharmacy and medical institutions may profit	125	12.5
6) It leads to a cleaner and more hygienic environment	50	5.0
7) Others	4	0.4
8) I don't know	177	17.7

Q20. If a patient requests the prescription of a drug that is not effective for treatment, the doctor may refuse to prescribe. For what kinds of diseases would you accept such policy? Please choose all that apply.

	n	%
1) Common cold	650	65.0
2) Headache	435	43.5
3) Abdominal pain	374	37.4
4) Backache	402	40.2
5) Others	28	2.8
6) Cannot accept for any disease	189	18.9

Q21. Have you ever heard of the word "placebo"?

	n	%
1) Yes, and I can explain the meaning of the word	201	20.1
2) Yes, I have heard of the word, but I cannot explain	160	16.0
3) I have not heard of the word / I don't know.	639	63.9
Total	1000	100
